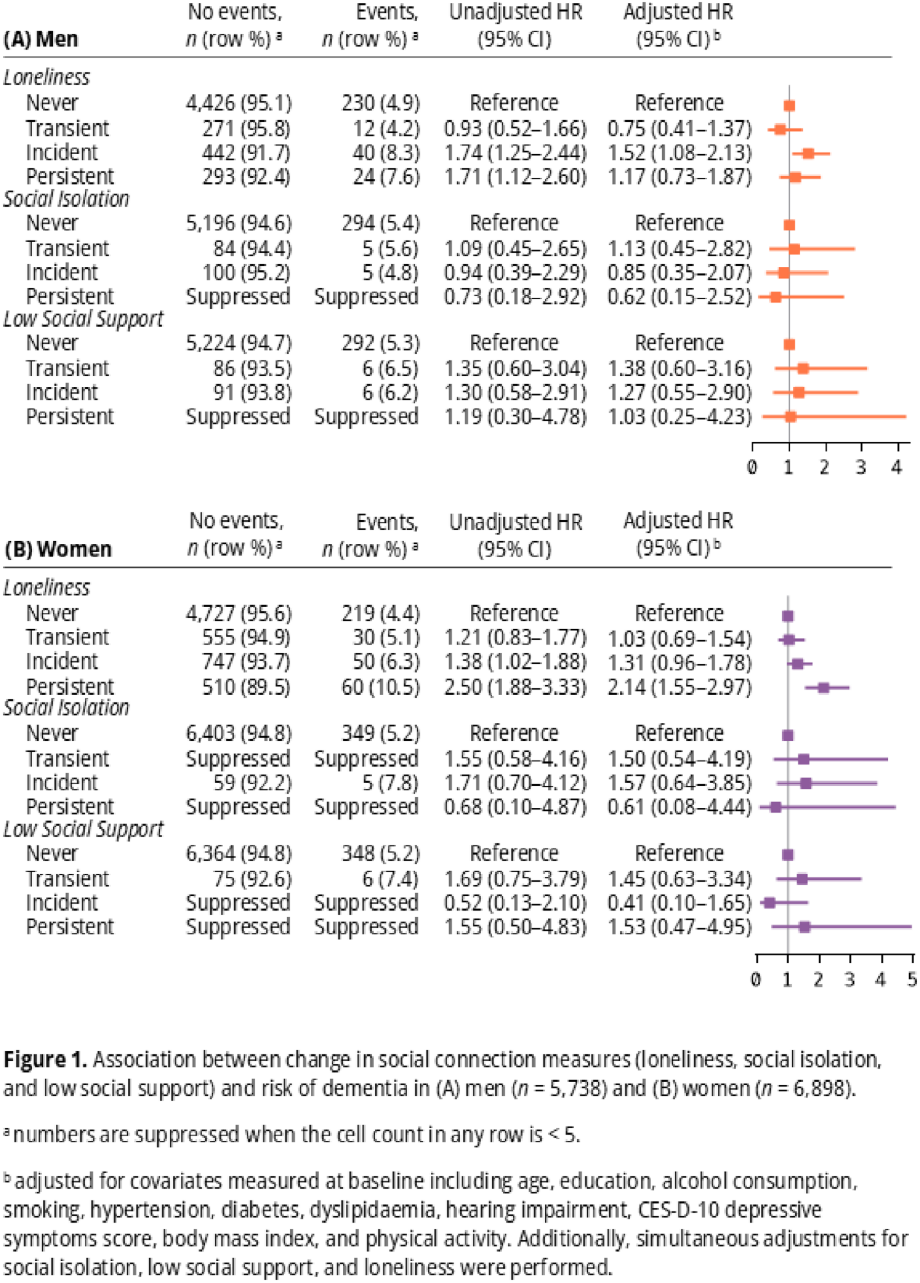# Changes in loneliness, social isolation, and social support and risk of dementia: a gender‐disaggregated analysis of community‐dwelling older adults

**DOI:** 10.1002/alz70860_100587

**Published:** 2025-12-23

**Authors:** Htet Lin Htun, Achamyeleh Teshale, Haoxiong Sun, Joanne Ryan, Alice Owen, Robyn L. Woods, Raj C. Shah, Trevor T.‐J. Chong, Rosanne Freak‐Poli

**Affiliations:** ^1^ Monash University, Melbourne, VIC, Australia; ^2^ Rush University Medical Center, Chicago, IL, USA; ^3^ Turner Institute for Brain and Mental Health & School of Psychological Sciences, Monash University, Clayton, VIC, Australia

## Abstract

**Background:**

Limited evidence exists regarding the gender‐specific impact of changes in loneliness, social isolation, and social support on incident dementia. This study aims to evaluate these changes and their associations with risk of dementia, separately for men and women.

**Method:**

We analysed data from over 12,000 community‐dwelling Australians aged 70+ years without significant cognitive impairment at enrolment, with a median follow‐up of over 8 years. Loneliness, social isolation, and social support were self‐reported at baseline and follow‐up (∼2 to 3 years later) and categorised as never, transient (present at baseline but not at follow‐up), incident (absent at baseline but present at follow‐up), or persistent (present at both). Dementia diagnosis followed *DSM‐IV* criteria, adjudicated by an expert panel. Gender‐disaggregated Cox proportional hazards regressions were conducted, adjusting for age and other known dementia risk factors.

**Result:**

At baseline, participants had a mean age of 75.2 years (± 4.3), with 54% being women. Overall, 81.1% of men and 71.7% of women reported never feeling lonely at baseline. Transient, incident, and persistent loneliness were experienced by 4.9%, 8.4%, and 5.5% of men, and 8.5%, 11.6%, and 8.3% of women, respectively. After adjusting for potential confounders, incident loneliness in men (HR: 1.52, 95% CI: 1.08‐2.13) and persistent loneliness in women (HR: 2.14, 95% CI: 1.55‐2.97) were associated with a greater dementia risk, compared to those who were never lonely (Figure 1).

**Conclusion:**

In later life, persistent loneliness among women and incident loneliness among men was associated with an increased risk of dementia. In this initially healthy cohort, very few participants reported social isolation and low social support at baseline and follow‐up, and neither was associated with dementia risk. Early identification of poor social connections, particularly loneliness, and timely intervention to prevent its progression into a chronic state may help maintain cognitive health and delay cognitive decline and dementia onset.